# Severe Tuberculosis Requiring Intensive Care: A Descriptive Analysis

**DOI:** 10.1155/2017/9535463

**Published:** 2017-01-30

**Authors:** Raquel Pacheco Duro, Paulo Figueiredo Dias, Alcina Azevedo Ferreira, Sandra Margarida Xerinda, Carlos Lima Alves, António Carlos Sarmento, Lurdes Campos dos Santos

**Affiliations:** ^1^Infectious Diseases Department, Centro Hospitalar de São João, Alameda Professor Hernâni Monteiro, Porto, Portugal; ^2^Instituto de Inovação e Investigação em Saúde (I3S), Grupo de I&D em Nefrologia e Doenças Infecciosas, Instituto Nacional de Engenharia Biomédica (INEB), Porto, Portugal

## Abstract

*Background*. This study aims to describe the characteristics of tuberculosis (TB) patients requiring intensive care and to determine the in-hospital mortality and the associated predictive factors.* Methods*. Retrospective cohort study of all TB patients admitted to the ICU of the Infectious Diseases Department of Centro Hospitalar de São João (Porto, Portugal) between January 2007 and July 2014. Comorbid diagnoses, clinical features, radiological and laboratory investigations, and outcomes were reviewed. Univariate analysis was performed to identify risk factors for death.* Results*. We included 39 patients: median age was 52.0 years and 74.4% were male. Twenty-one patients (53.8%) died during hospital stay (15 in the ICU). The diagnosis of isolated pulmonary TB, a positive smear for acid-fast-bacilli and a positive PCR for* Mycobacterium tuberculosis* in patients of pulmonary disease, severe sepsis/septic shock, acute renal failure and Multiple Organ Dysfunction Syndrome on admission, the need for mechanical ventilation or vasopressor support, hospital acquired infection, use of adjunctive corticotherapy, smoking, and alcohol abuse were significantly associated with mortality (*p* < 0.05).* Conclusion*. This cohort of TB patients requiring intensive care presented a high mortality rate. Most risk factors for mortality were related to organ failure, but others could be attributed to delay in the diagnostic and therapeutic approach, important targets for intervention.

## 1. Introduction

Tuberculosis (TB) remains a major public health problem and ranks alongside the human immunodeficiency virus (HIV) as a leading cause of death worldwide [1]. In 2014, the global burden of* Mycobacterium tuberculosis* infection included 9.6 million new cases (of whom 12% were HIV-positive) with 1.5 million deaths from the disease [2]. In Portugal, the incidence rate in 2014 was of 20.0 cases/100.000 population, with 13.3% of the patients being HIV-infected (the most frequent comorbidity) [3]. Although the trend in the last years has been of a steady decline in TB incidence, there is still an important association with the most vulnerable groups (homeless, foreign born, drug addicts, and prisoners), often with poorer access to health care, preventing an early diagnosis and treatment [3].

In 2012, the mortality rate of tuberculosis in Portugal was 2.0/100.000 habitants, with significantly higher values for the elderly; more than 1000 patients with TB are still admitted in the hospital every year, with a lethality rate of 12.4% [4]. Both early diagnosis and early treatment are crucial for TB control, as delayed diagnosis and/or treatment is associated with higher mortality rates and may lead to severe clinical forms of the disease [5–8]. Moreover, late diagnosis and treatment are major contributors to the dissemination of the disease, with dramatic public health implications.

Severe TB that requires Intensive Care Unit (ICU) admission generally presents as respiratory failure, and, despite the availability of effective therapies, mortality rates remain between 15.5 and 65.9% [5–7, 9–13]. Risk factors associated with mortality vary between studies, but the most frequent are the presence of organ failure, respiratory insufficiency requiring mechanical ventilation, sepsis, the presence of other infections, and delayed treatment [6, 7, 9–12].

In the early studies, HIV infection was an independent risk factor for mortality in the ICU; in fact, it was included in the Simplified Acute Physiologic Score (SAPS) II score [14]. Coinfection of HIV and TB is not only frequent but also associated with higher mortality and poorer outcomes [2, 3, 15], often presenting with atypical TB and potentially delayed treatment due to diagnostic difficulties and impaired access to healthcare [16]. However, for TB patients in the ICU, HIV has not been a consistent risk factor for mortality [9–11].

The purpose of this study was to describe the characteristics of TB patients requiring intensive care and to determine in-hospital mortality rate and predictive factors for mortality in an Intensive Care Unit in a tertiary hospital in Portugal.

## 2. Material and Methods

We retrospectively included all adult patients (>18 years old) admitted to the ICU of the Infectious Diseases Department of Centro Hospitalar de São João, Porto, Portugal (a Level III ICU according to ESICM classification [17]), between January 2007 and July 2014. Using the hospital database (where all patients admitted to the hospital are classified using the International Disease Classification, version 10), we identified those with suspected TB and reviewed their medical charts and records. All patients with active TB were included. Patients admitted to the ICU who had received <3 months of antituberculosis treatment were also included, as TB or antituberculous treatment could not be ruled out as a reason for ICU admission. Of note, during this period, a total of 1273 patients were admitted in Centro Hospitalar de São João (infirmary and intensive care) with TB as a major clinical diagnosis (on average 165.9 patients/year).

Data on comorbid diagnoses, clinical features, radiological and laboratory investigations, and outcomes were collected after medical charts review.

We defined active TB as (1)* M. tuberculosis*-positive culture, (2) presence of acid-fast bacilli (AFB) on Ziehl-Neelsen staining plus clinical response to treatment, (3) histological results plus clinical response to antituberculous treatment, (4) positive polymerase chain reaction (PCR) for* M. tuberculosis* with clinical response to antituberculous treatment, and (5) presence of clinical, epidemiological, and radiographic findings compatible with TB and with clinical response to therapy.

The following data were collected in a standardized questionnaire: demographic data (sex, age), smoking status, alcoholism, injection drug use, presence of comorbidities, clinical form of TB (patients were classified as having isolated pulmonary involvement, pulmonary and extrapulmonary involvement, or isolated extrapulmonary involvement), symptoms at admission, methods of diagnosis, prior TB treatment, interval from hospital admission to ICU admission (early ICU admission defined has direct admission or transfer to the ICU within 48 hours after hospital admission), reasons for ICU admission, Acute Physiologic and Chronic Health Evaluation (APACHE) II and SAPS II scores, presence of other acute diseases on admission, need for ventilatory or hemodynamic support on ICU admission and during ICU stay, length of mechanical ventilation, ARDS, ECMO, renal replacement therapy, length of ICU and hospital stay, hospital acquired infections, interval from hospital admission to treatment initiation (early treatment defined has beginning of antituberculous treatment within the first 3 days of hospital admission), drug regimen, chest X-ray on admission (number of quadrants involved; presence of cavities; miliary pattern), laboratory investigations on admission (white cell count, hemoglobin, albumin, total protein, C-reactive protein, and renal function), survival at ICU discharge, hospital discharge, and 12 months after ICU admission.

Culture samples for TB diagnosis included sputum, tracheal and nasogastric aspiration, bronchoalveolar lavage, lymph node aspiration, cerebrospinal fluid, pericardial fluid, peritoneal fluid, urine, pleural fluid, and blood. All samples were cultured in liquid medium incubated in an automated system (BACTEC MGIT 960®, mycobacterial growth indicator tube system). Drug susceptibility testing was performed on all culture positive samples. Drug resistance was defined as either single or poly-drug resistance to two or more anti-TB drugs. PCR for* M. tuberculosis *in our hospital is an* “in-house”* technique.

A diagnosis of respiratory failure was made after the determination of arterial oxygen pressure (PaO_2_) level <60 mmHg or arterial oxygen saturation (SaO_2_) <90% when breathing room air, with or without elevation of arterial PCO_2_. Ventilator-associated pneumonia (VAP) was diagnosed based on the American Thoracic Society (ATS) criteria (ATS 2005). Multiple organ dysfunction syndrome (MODS) was defined as mild to severe dysfunction of two or more organs [18]. Anemia was defined by hemoglobin levels <13.5 g/dL for males and <12.0 g/dL for females. Sepsis and sepsis-related conditions were defined according to the criteria proposed by the American College of Chest Physicians/Society of Critical Care Medicine [19].

The protocol was submitted to the Ethics Committee of our hospital and an approval was obtained.

### 2.1. Statistical Analysis

Data were analyzed using SPSS® v22.0 (Statistical Package for the Social Sciences, Chicago, Illinois) and GraphPad® Prism 6. Continuous variables were expressed as median and interquartile range (IQR) and were compared using Mann–Whitney* U* test. Categorical variables were expressed as proportion and were compared using Chi-square test or Fisher's exact test (as appropriate). Data were compared between survivors and patients who died during hospital stay. A univariate analysis was performed to identify risk factors for death. The Kaplan-Meier method was used to analyze survival. A two-sided *p* value <0.05 was considered statistically significant for all analysis.

## 3. Results

### 3.1. Patient Characteristics

Of the 40 patients reviewed for active TB, we excluded one case of renal TB due to intravesical immunotherapy with bacilli Calmette-Guérin (BCG) for bladder carcinoma. The epidemiological and clinical characteristics of the 39 patients included in this analysis are shown in Table 1.

The median age was 52.0 years (IQR 25.0); 29 (74.4%) were male. Twenty-nine patients (74.4%) had at least one comorbidity. The most frequent was HIV infection (15 patients, 38.5%); the median of CD4 counts of these patients was 50.5 cells/*μ*L (IQR 209.5); only one patient was receiving highly active antiretroviral therapy (HAART) at the time of hospitalization; the remaining had either previously abandoned therapy (9 patients) or were diagnosed on admission (5 patients). Two patients were receiving immunosuppressive therapy due to autoimmune disorders: pemphigus vulgaris with corticosteroids and mycophenolate-mofetil, and rheumatoid arthritis with corticosteroids and rituximab. A history of previous TB was present in 5 patients (all had completed therapy more than 5 years previous to hospital admission).

### 3.2. Disease Presentation and Diagnosis

Almost all patients (94.9%) reported symptoms prior to hospital admission; the most common (71.8%) were constitutional symptoms (fever, weight loss, night sweats, asthenia, and/or anorexia), followed by respiratory symptoms (64.1%; productive cough, dyspnea, and hemoptysis).

Table 2 shows data regarding diagnosis, microbiological data, and radiological findings. Twenty-eight patients (71.8%) presented with isolated pulmonary TB; 8 patients (20.5%) with involvement of pulmonary and extrapulmonary organs (two genitourinary and peritoneal, two meningeal, one genitourinary, one meningeal and lymphatic, one pleural, and one renal and hematological); and three patients (7.7%) with isolated extrapulmonary TB (meningeal, lymphatic, and pericardial, one case each).

All patients with pulmonary TB (either isolated or with an extrapulmonary component, *n* = 36) collected at least one respiratory sample for microbiological examination (either sputum, bronchial aspirate, or bronchoalveolar lavage). Of these, 25 (69.4%) had at least one positive smear for AFB, 30 (83.3%) had positive culture, and 19 (of the 31 tested; 61.3%) positive PCR for* M. tuberculosis* (90.0% in smear-positive patients).

Cultures of respiratory samples were positive in all patients (*n* = 8) with pulmonary and extrapulmonary; 7 of those also had* M. tuberculosis* growth in nonrespiratory samples. All isolated extrapulmonary TB patients had negative cultures and so diagnosis was made on purely clinical grounds.

Cultures to* M. tuberculosis* were negative in 9 patients (23.1%), 5 of which were HIV-infected; one had AFB in a histological sample of a lymphatic biopsy (that was not cultured) and in the remaining 8 patients TB diagnosis was based solely on clinical, epidemiological, and radiographic findings. They all started antituberculous therapy with clinical improvement except one patient that died (an HIV-infected patient with the simultaneous diagnosis of pneumocystosis that developed VAP caused by a MDR* P. aeruginosa*). Among the 30 patients with cultures positive for* M. tuberculosis*, the susceptibility tests showed single-drug resistance in three patients (2 to streptomycin and 1 to isoniazid), with no poly-drug or MDR resistance detected; of these, only the patient with isoniazid resistance died.

Compared to non-HIV-infected patients, the HIV-infected group was more likely to present with respiratory symptoms (86.7% versus 50.0%, *p* = 0.02) and to be admitted in the ICU for respiratory failure (73.3% versus 37.5%, *p* = 0.029). When analyzing patients with pulmonary TB, the HIV-infected group was less likely to have a positive smear for AFB (46.7% versus 75.0%, *p* = 0.02), and none presented cavities on X-ray (0% versus 25.0%, *p* = 0.03).

### 3.3. ICU Presentation and Management

Table 2 also shows ICU characteristics at admission and complications, including a comparison between survivors and nonsurvivors.

Acute respiratory failure due to TB was the primary cause of ICU admission in 20 patients (51.3%). Twenty patients (51.2%) were admitted early in the ICU; the median number of days until ICU admission was 4 days (IQR 3).

Concurrent nontuberculous infection at the time of admission was diagnosed in 12 patients (30.8%; six of these were HIV-infected), and some patients presented more than one concurrent infection (*n* = 5). Pneumonia was the main infection recorded (*n* = 6), followed by pneumocystosis (*n* = 3). The other infections were endocarditis, Mediterranean spotted fever, aspergillosis (histological diagnosis of a tracheal biopsy), Fournier's gangrene, cerebral toxoplasmosis, and pulmonary* Mycobacterium avium complex* (positive cultural bronchoalveolar lavage and blood culture in a HIV-infected patient). Five patients presented with concomitant noninfectious acute diseases: drug overdose, B cell lymphoma, gastric ulcer perforation, upper gastrointestinal bleeding, and decompensated cardiac failure.

Thirty-three patients (84.6%) were already on or began antituberculous treatment in the ICU; 24 patients (61.5%) were on therapy by the third day after ICU admission. Twelve patients started antituberculous therapy purely based on clinical suspicion (this is, with no positive smear or PCR testing); eight started therapy in the ICU (five of which within 3 days) and cultures were later positive in four of these patients. They all began a four-drug regimen including rifampicin; there were only two cases described of hepatotoxicity and two cases of allergic reaction to rifampicin. The remaining 6 patients began antituberculous treatment after ICU discharge; one of these patients died during hospital stay. Corticotherapy was used as adjunctive therapy in five patients, those with the diagnosis of either meningeal or pericardial tuberculosis.

There were no significant differences between HIV-positive and negative patients in terms of ICU status at admission (other than the described differences relating to reason for ICU admission) or posterior management.

### 3.4. Outcome and Risk Factors for Mortality

**Figure 1:**
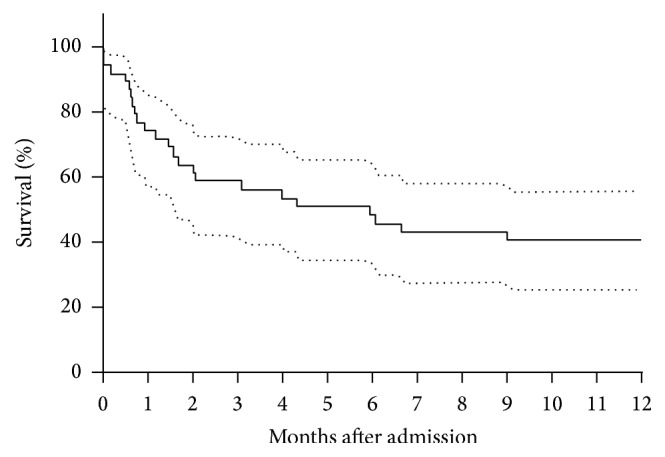
Twelve months' survival using Kaplan-Meier (*n* = 39).

ICU mortality was 38.5% (15 of 39 patients) and in-hospital mortality 53.8%. The median in-hospital survival time of patients who died was 55.4 days (IQR 59.5). The Kaplan-Meier survival curve is shown in Figure 1; the probability of survival at 12 months after ICU admission was 41.0% (IC 95% 25.7–55.7).

The median APACHE II score was 26.0 (IQR 15.75) and the median SAPS II score was 55.0 (IQR 27.5), with a predicted mortality of 57% and 59%, respectively; it was higher in HIV-infected patients: 29.5 (IQR 10.75) and 65 (IQR 27.5), with a predicted mortality of 68% and 78%, respectively.

The median duration of hospital stay was 42.0 days (IQR 47), and the median duration of ICU care 12.0 days (IQR 25). Due to death, the duration of hospital stay was shorter in the nonsurvivor group (35.0 days [IQR 60] versus 43 days [IQR 43.5]; however, ICU stay was longer in the nonsurvivor group (21.0 days [IQR 40.5] versus 6.5 days [IQR 8.75]).

Risk factors for in-hospital mortality (using univariate analysis) are summarized in Tables 1 and 2. Some risk factors observed were related to TB while others were related to organ failure.

The following factors related to TB were significantly associated with in-hospital mortality: diagnosis of isolated pulmonary TB (*p* = 0.037, OR = 5.667 [1.034–22.293]); in pulmonary TB, positive smear for AFB (*p* = 0.034, OR 5.667 [1.178–27.254]) and positive PCR for* M. tuberculosis* in respiratory samples (*p* = 0.008, OR = 8.4 [1.6–44.104]). Of note, patients with the diagnosis of isolated pulmonary TB presented higher SAPS II and APACHE II scores (*p* = 0.002 and *p* = 0.024) and were more likely to need ventilatory and hemodynamic support (resp., 85.7% versus 45.4%, *p* = 0.17; and 67.8% versus 18.2%, *p* = 0.05).

The following factors not related to TB were significantly associated with in-hospital mortality: when present on ICU admission, severe sepsis/septic shock (*p* = 0.049, OR = 8.5 [0.931–77.598]), mechanical ventilation (*p* = 0.041, OR = 4.25 [1.019–17.729]), vasopressor support (*p* = 0.02, OR = 5.5 [1.219–24.813]), and acute renal failure (*p* = 0.049, OR = 8.5 [0.931–77.593]); during ICU stay, the development of MODS (*p* = 0.028, OR = 6.0 [1.090–33.016]), the need for mechanical ventilation (*p* = 0.002, OR = 20.0 [2.192–182.442]) or vasopressor infusion (*p* < 0.001, OR = 30.0 [5.261–171.062]), and the development of hospital acquired infection (*p* = 0.028, OR = 6.0 [1.090–33.016]); of the baseline patient characteristics, smoking (*p* = 0.041, OR = 4.545 [1.008–20.507]) and alcohol abuse (*p* = 0.049, OR = 8.5 [0.0931–77.598]).

Regarding treatment, early initiation of antituberculous therapy (by the third day of ICU admission) was not associated with survival (*p* = 0.420). However, adjunctive corticotherapy (only used in meningeal or pericardial disease) was associated with survival (*p* = 0.015).

It should be noted that HIV positivity was not a risk factor for mortality; in fact, in-hospital mortality observed was lower in the HIV-infected group (40.0% versus 62.5%, *p* = 0.17). However, SAPS II score (which considers HIV infection an important mortality predictor) was significantly higher in HIV-infected patients (*p* = 0.032); although higher in the HIV-infected group, this difference did not reach statistical significance with APACHE II score. Regarding HAART, of the 14 HIV-infected patients not on treatment, only one started therapy during that hospital admission (not in the ICU). The remaining eight patients who survived started HAART in the ambulatory setting; six were alive 12 months after ICU admission.

## 4. Discussion

This retrospective analysis of 39 TB patients requiring intensive care found a high in-hospital mortality rate (53.8%), within the range of published data [7, 9–13, 20–22] and in agreement with the predicted mortality. It is considerably higher than the mortality rate of all TB patients requiring hospital admission in our country (12.4%) [4]. We aimed to evaluate the risk factors associated with mortality.

In this cohort the median age was 52.0 years, higher than the majority of published series where it ranged from 36.6 to 47.8 years [5, 7–11]; this may in part account for the high mortality, as age has been recognized as a risk factor [6, 10]. There was a clear male predominance, in agreement with the majority of the published data [5, 7, 9–12]. Patients with TB requiring ICU care have high rates of comorbidities [9, 10, 20, 23], as in this sample.

TB usually affects the lungs but may present in almost any organ system [24]. As in other studies [9–11, 20], there was a predominance of pulmonary TB and acute respiratory failure was the main reason for ICU admission. The majority presented with severe radiographic alterations (more than two-thirds with involvement of three or four pulmonary lobes), a possible reflection of a protracted disease process; however, it did not significantly impact the mortality rate, unlike in other studies [7, 12].

The second most frequent reason for ICU admission was severe sepsis/septic shock, with most patients presenting with MODS and a higher mortality compared to other causes of ICU admission. This was also the case of other studies [7, 10, 12], reflecting the severity of this presentation.

As in previous studies [6–8, 10, 11], organ failure negatively affects prognosis and is associated with higher mortality rates. The need for respiratory and/or vasopressor support and the presence of acute renal failure and of multiple organ dysfunction were associated with higher mortality. The severity scores were significantly higher in patients that did not survive.

The development of hospital acquired infections has been long described as a negative prognostic factor in ICU patients. In this sample, the proportion of patients that developed at least one hospital acquired infection was not high (28.2%) compared with other studies [9–11], but the development of such complication was associated with higher mortality rates.

Pulmonary TB patients with a positive smear for AFB and a positive PCR for* M. tuberculosis* presented higher mortality rates, and a similar trend could be seen in those with positive cultural examination. This was also reported by Valade et al. [22] whilst Silva et al. [9] reported the opposite, with smear-positive sputum as a protective factor (in view of more timely diagnoses). It could be argued that there was a higher mycobacterial burden in these patients, probably representing a longer disease process.

Microbiological diagnosis of tuberculosis has improved over time but is still not optimal and especially not as expeditious as desired, as it takes from 2 to 8 weeks for* M. tuberculosis* to grow in culture, with faster results in liquid medium [20, 25]. Molecular biology techniques, such as PCR, are useful tools for a quicker diagnosis of TB, with implications on treatment initiation. In our hospital, the available PCR is an* “in-house”* technique, with a global sensitivity of 61.2%, a very good performance on positive smear patients (90.0%) but a lower sensitivity in negative ones (9.1%). This has been documented extensively in the literature [26].* GeneXpert®* is a new fast PCR technique with better performance in negative smear sputum [27], not available in our hospital. There is still little data on the performance and mortality impact of this technique in the intensive care setting, although a clinical trial in South Africa [28] shows better performance when compared with smear for AFB alone.

TB is a treatable disease and it has been documented that a proactive approach with timely intervention is required in the treatment of critically ill TB patients; in fact, later onset of treatment has been associated with a higher mortality [5, 7, 8]. As such, treatment should be initiated before microbiological confirmation, especially in the ICU patients [20].

Actually, an important proportion of patients (23.1%, more than half being HIV-infected) had, in the end, only a presumptive diagnosis for TB, with no isolation of* M. tuberculosis* in culture; they all started treatment and only one died. This is not new to the diagnostic and treatment approach to tuberculosis [7, 9, 11, 12], making it clear that definite TB diagnosis is not always simple: if the clinical suspicion is high, treatment should be started without delay (always collecting appropriate samples for culturing), and if there is an improvement it should be continued even if culture results are negative [29].

As already stated, delayed treatment initiation has been associated with higher mortality [5, 7, 8]. In agreement with this, we observed a lower mortality rate (although not statistically significant) in the group of patients that started antituberculous therapy within 3 days after ICU admission. A high percentage of patients (84.6%) started therapy while in the ICU, with a four-drug treatment; the rate of hepatotoxicity and hypersensitivity reactions was lower than the one described in other studies.

Adjunctive corticotherapy was used in patients with meningeal and pericardial disease (five patients), none of which died (*p* = 0.015). Over time, there has been considerable debate regarding adjunctive corticotherapy in the setting of TB disease; currently, only meningeal and pericardial diseases are formal indication for such therapy according to major guidelines [30–32]. However, patients requiring intensive care admission due to TB disease may be a particular subset in whom there is some data suggesting a mortality benefit with this therapy [33, 34]. More studies are needed in order to clarify this issue.

As a known risk factor for TB development, HIV infection was a prevalent comorbidity in this analysis (38.5%), within the range described in other studies (7 to 68.7% [5, 9–11]). The diagnostic difficulties are higher in this population, with lower probability of a positive smear for AFB or a cultural examination of respiratory samples; this has been well documented and correlated with the paucibacilar nature of TB in this subset of patients [35]. Noticeably, HIV infection was not a risk factor for mortality. In fact, the mortality observed was lower in the HIV-infected group (40% versus 62.5% in non-HIV-infected patients). This lack of association with mortality has also been noted in other studies [9–11], with HIV infection presenting as a risk factor for mortality only in older studies [5]. This is in line with the trend of improved ICU survival of HIV-infected patients observed recently [36, 37].

Nothing has impacted the survival of HIV-infected patients as HAART [38]. However, in the intensive care environment, there are several specific issues: difficulties with medication delivery, overlapping toxicities, potential interactions, the risk of immune reconstitution inflammatory syndrome (IRIS), and erratic drug absorption [39, 40]. Moreover, coinfection of TB and HIV and timing for HAART initiation are an even more complex subject, aggravating the previously mentioned issues. It is currently advocated an earlier introduction of HAART in patients with severe immunodepression but a relative delay in those not so severely immunodepressed [41–43]. In this study, HAART was only initiated on one of the 14 nontreated HIV-infected patients. Many different reasons contributed to the postponing of HAART initiation: poor adherence (nine patients had already previously abandoned treatment), drug toxicities, and the potential of drug interactions were the most relevant.

This study has some limitations: it is a retrospective single center study with a low sample size (underpowering the analysis). We could not evaluate duration of symptoms before hospital admission; however, patients with active tuberculosis admitted to ICUs have advanced disease and surely we are facing late diagnosis with a long disease process. Despite these limitations, it is a real setting based study and, to our knowledge, it is the first study in Portugal that described TB cases and their outcomes in patients requiring intensive care.

## 5. Conclusions

TB is still an important disease in Portugal, despite the fact that much has been done in recent years to improve diagnostic and treatment procedures, with a clear improvement in the scores analyzed [3]. The present study found a high mortality rate of tuberculosis patients requiring Intensive Care Unit admission. Most risk factors for mortality are related to severity of organ failure, and others (such as nosocomial infections) were actually related to intensive care procedures. However, many can be easily attributed to delay in the diagnostic and therapeutic approach. As such, measures aimed at promoting early diagnosis and treatment can contribute to better overall outcomes, at the same time breaking the chain of transmission and consequent burden of the disease.

## Figures and Tables

**Table 1:** Patient characteristics, including comparison between survivors and nonsurvivors.

	Nonsurvivors *n* = 21 (53.8%)	Survivors *n* = 18 (46.2%)	Total *n* = 39	*p* value
Age (years)	54.0 (30.0)	47.0 (25.8)	52.0 (25.0)	0.076
Sex				0.141
Female, *n* (%)	3 (30.0)	7 (70.0)	10 (25.6)	
Male, *n* (%)	18 (62.1)	11 (37.9)	29 (74.4)	
Race				0.206
White race, *n* (%)	21 (56.8)	16 (43.2)	37 (94.9)	
Black race, *n* (%)	0	2 (100)	2 (5.1)	
Homelessness	2 (50.0)	2 (50.0)	4 (10.3)	1
Comorbidities				
Presence of any comorbidity, *n* (%)	18 (62.1)	11 (37.8)	29 (74.4)	0.141
Immunodeficiency				
HIV infection, *n* (%)	6 (40.0)	9 (60.0)	15 (38.5)	0.170
Immunosuppressive therapy, *n* (%)	0	2 (100)	2 (5.1)	0.206
Cancer, *n* (%)	1 (100)	0	1 (2.6)	1
Smokers, *n* (%)	10 (76.9)	3 (23.1)	13 (33.3)	**0.041**
Alcohol abuse, *n* (%)	7 (87.5)	1 (12.5)	8 (20.5)	**0.049**
Drug addiction, *n* (%)	4 (44.4)	5 (55.6)	9 (23.1)	0.706
COPD^1^, *n* (%)	6 (75.0)	2 (25.0)	8 (20.5)	0.247
Silicosis, *n* (%)	1 (100)	0	1 (2.6)	1
Previous TB, *n* (%)	3 (60.0)	2 (40.0)	5 (12.8)	0.247
Diabetes *mellitus*, *n* (%)	1 (100)	0	1 (2.6)	1
Chronic renal disease, *n* (%)	0	1 (100)	1 (2.6)	0.462
Chronic hepatic disease, *n* (%)	1 (50.0)	1 (50.0)	2 (5.1)	1
Undernutrition, *n* (%)	7 (70.0)	3 (30.0)	10 (25.6)	0.290

^1^COPD: chronic obstructive pulmonary disease.

**Table 2:** Risk factors for in-hospital mortality using univariate analysis.

	Nonsurvivors *n* = 21 (53.8%)	Survivors *n* = 18 (46.2%)	Total *n* = 39	*p* value
Diagnosis				
Isolated pulmonary TB, *n* (%)	18 (64.3)	10 (35.7)	28 (71.8)	**0.037**
Pulmonary and extrapulmonary TB, *n* (%)	2 (25.0)	6 (75.0)	8 (20.5)	0.112
Isolated extrapulmonary TB, *n* (%)	1 (33.3)	2 (66.7)	3 (7.7)	0.586
Pulmonary TB (*n* = 36)				
Microbiological data				
Positive smear for AFB, *n* (%)	17 (68.0)	8 (32.0)	25 (69.4)	**0.034**
Positive cultural examination, *n* (%)	19 (63.3)	11 (36.7)	30 (83.3)	0.069
Positive PCR (*n* = 31), *n* (%)	14 (73.7)	5 (26.3)	19 (61.3)	**0.008**
Radiological findings				
Miliary radiological pattern, *n* (%)	8 (61.5)	5 (38.4)	13 (36.1)	0.587
Cavitary disease, *n* (%)	4 (6.7)	2 (33.3)	6 (16.7)	0.672
Multilobar involvement (≥3), *n* (%)	16 (57.1)	12 (42.9)	28 (77.8)	1
ICU admission				
Physiological score				
APACHE II, median (IQR)	30.0 (12.75)	20.5 (17.00)	26.0 (15.75)	**0.030**
SAPS II, medina (IQR)	58.0 (23.5)	42.5 (38.50)	55.0 (27.5)	**0.014**
Early ICU admission, *n* (%)	8 (40.0)	12 (60.0)	20 (51.3)	0.075
Reasons for ICU admission				
Respiratory failure, *n* (%)	10 (50.0)	10 (50.0)	20 (51.3)	0.621
Severe sepsis/septic shock, *n* (%)	7 (87.5)	1 (12.5)	8 (20.5)	**0.049**
Decreased consciousness, *n* (%)	0	2 (100)	2 (5.1)	0.206
Post-CPR, *n* (%)	3 (75.0)	1 (25.0)	4 (10.3)	0.609
Post-surgical procedure, *n* (%)	1 (20.0)	4 (80.0)	5 (12.8)	0.162
Physiological support				
Mechanical ventilation, *n* (%)	17 (65.4)	9 (34.6)	26 (66.7)	**0.041**
Vasopressor infusion, *n* (%)	11 (78.6)	3 (21.4)	14 (35.9)	**0.02**
Acute renal failure, *n* (%)	7 (87.5)	1 (12.5)	8 (20.5)	**0.049**
MODS, *n* (%)	9 (81.8)	2 (18.2)	11 (28.2)	**0.028**
Laboratory results				
Hb, median (IQR)	10.8 (2.6)	11.3 (5.0)	10.85 (3.6)	0.794
WBC, median (IQR)	10290 (6665)	6210 (6758)	8290 (7850)	0.076
C-reactive protein, median (IQR)	112 (82.85)	66.05 (118.97)	96.5 (103.4)	0.143
Creatinine, median (IQR)	0.66 (0.43)	0.7 (0.21)	0.675 (0.315)	0.663
Total protein, median (IQR)	53.6 (14.2)	56.6 (7.38)	54.8 (12.5)	0.069
Albumin, median (IQR)	20.3 (7.7)	23.5 (9.12)	20.9 (8.0)	0.132
Presence of other infections/acute diseases at admission, *n* (%)	7 (41.2)	10 (58.8)	17 (43.6)	0.163
Management and complications in the ICU				
Mechanical ventilation, *n* (%)	20 (69.0)	9 (31.0)	29 (74.4)	**0.002**
Days of mechanical ventilation, median (IQR)	24.0 (40.0)	7.0 (14.5)	17.0 (39.00)	0.14
ARDS, *n* (%)	6 (85.7)	1 (14.3)	7 (17.9)	0.098
ECMO, *n* (%)	2 (100)	0	2 (5.1)	0.49
Vasopressor infusion, *n* (%)	18 (85.7)	3 (14.3)	21 (53.8)	**<0.001**
Renal replacement therapy, *n* (%)	3 (100)	0	3 (7.7)	0.235
Hospital acquired infections, *n* (%)	9 (81.8)	2 (18.2)	11 (28.2)	**0.028**
VAP, *n* (%)	6 (75.0)	2 (25.0)	8 (20.5)	0.247
Catheter related infection, *n* (%)	3 (100)	0	3 (7.7)	0.235
Antituberculous therapy initiated in the ICU, *n* (%)	20 (60.6)	13 (39.4)	33 (84.6)	0.077
Within 3 days of ICU admission, *n* (%)	13 (54.2)	11 (45.8)	24 (72.7)	0.420
After the third day of ICU admission, *n* (%)	7 (77.8)	2 (22.2)	9 (27.3)	0.420
Days until therapy initiation, mean (IQR)	0 (4)	0 (2)	0 (4)	0.545
Adjunctive corticotherapy, *n* (%)	0	5 (100)	5 (12.8)	**0.015**
